# Critical care outcomes in decompensated cirrhosis: a United States national inpatient sample cross-sectional study

**DOI:** 10.1186/s13054-024-04938-8

**Published:** 2024-05-07

**Authors:** Spencer R. Goble, Abdellatif S. Ismail, Jose D. Debes, Thomas M. Leventhal

**Affiliations:** 1 Department of Medicine, Hennepin Healthcare, 730 South 8th Street, Minneapolis, MN 55415 USA; 2 Department of Internal Medicine, University of Maryland Medical Center Midtown Campus, 827 Linden Ave, Baltimore, MD 21201 USA; 3 Department of Medicine, University of Minnesota, Mayo Memorial Building, MMC 250, 420 Delaware Street S.E., Minneapolis, MN 55455 USA; 4 Division of Gastroenterology, Hepatology, and Nutrition, University of Minnesota, MMC 36, 420 Delaware Street S.E., Minneapolis, MN 55455 USA

**Keywords:** Palliative care, Intensive care units, Patient care planning

## Abstract

**Background:**

Prior assessments of critical care outcomes in patients with cirrhosis have shown conflicting results. We aimed to provide nationwide generalizable results of critical care outcomes in patients with decompensated cirrhosis.

**Methods:**

This is a retrospective study using the National Inpatient Sample from 2016 to 2019. Adults with cirrhosis who required respiratory intubation, central venous catheter placement or both (n = 12,945) with principal diagnoses including: esophageal variceal hemorrhage (EVH, 24%), hepatic encephalopathy (58%), hepatorenal syndrome (HRS, 14%) or spontaneous bacterial peritonitis (4%) were included. A comparison cohort of patients without cirrhosis requiring intubation or central line placement for any principal diagnosis was included.

**Results:**

Those with cirrhosis were younger (mean 58 vs. 63 years, *p* < 0.001) and more likely to be male (62% vs. 54%, *p* < 0.001). In-hospital mortality was higher in the cirrhosis cohort (33.1% vs. 26.6%, *p* < 0.001) and ranged from 26.7% in EVH to 50.6% HRS. Mortality when renal replacement therapy was utilized (n = 1580, 12.2%) was 46.5% in the cirrhosis cohort, compared to 32.3% in other hospitalizations (*p* < 0.001), and was lowest in EVH (25.7%) and highest in HRS (51.5%). Mortality when cardiopulmonary resuscitation was used was increased in the cirrhosis cohort (88.0% vs. 72.1%, *p* < 0.001) and highest in HRS (95.7%).

**Conclusions:**

One-third of patients with cirrhosis requiring critical care did not survive to discharge in this U.S. nationwide assessment. While outcomes were worse than in patients without cirrhosis, the results do suggest better outcomes compared to previous studies.

**Supplementary Information:**

The online version contains supplementary material available at 10.1186/s13054-024-04938-8.

## Background

Cirrhosis is a major cause of global morbidity and mortality with annual deaths and hospitalizations continuing to rise [1–5]. In the United States (U.S.) alone, chronic liver disease accounts for nearly 700,000 annual hospitalizations and 44,000 annual deaths [6, 7]. Acute decompensation events in cirrhosis, such as gastrointestinal hemorrhage and hepatic encephalopathy, are associated with high rates of both multiorgan dysfunction and short-term mortality [8–11]. To optimally manage these life-threatening complications, critical care is sometimes necessary. Indeed, 10% of patients hospitalized for a complication of cirrhosis require intensive care unit (ICU) admission and 3% of all ICU admissions occur for patients with cirrhosis [12, 13]. Historically, outcomes have been considered very poor for patients with complications of decompensated cirrhosis requiring ICU-level care [10, 14–16]. Reported ICU mortality rates for patients with cirrhosis have ranged from 29 to 87% [4, 8, 13, 17–20]. While recent studies have suggested that outcomes in critical care for decompensated cirrhosis may be improving, the generalizability of available data is limited by differences in studied populations and institutions along with low sample sizes and overall outcomes in the U.S. are still unclear [2, 13, 21].

Understanding prognosis is important for providers as it informs clinical care and can help with proper stratification in the management of these patients. It also allows for informed discussions with patients and their families about the utility of aggressive care [22]. In an attempt to objectively demonstrate critical care outcomes in those with cirrhosis admitted to an ICU and allow for future assessments in outcome trends, we utilized the U.S. National Inpatient Sample (NIS) to characterize decompensation type, interventions utilized, and in-hospital mortality.

## Methods

### Study design and database description

This is a retrospective, cross-sectional study that utilized the NIS to analyze hospitalizations in the U.S. 2016–2019. The NIS is a database that was developed for the Healthcare Cost and Utilization Project (HCUP). It is an all-payer database that approximates a 20-percent stratified sample of U.S. community hospital admissions. After weighting, the NIS can characterize approximately 35 million annual hospitalizations. Basic patient demographic data along with hospital data such as hospital size and region are both available for individual hospitalizations within the NIS. The NIS contains a single principal discharge diagnosis which is considered to be the diagnosis chiefly responsible for the admission. Procedures completed during the hospitalization are also available within the NIS. The NIS is a de-identified database that is publicly available and Institutional Review Board approval was waived.

### Study sample and variables

**Fig. 1:**
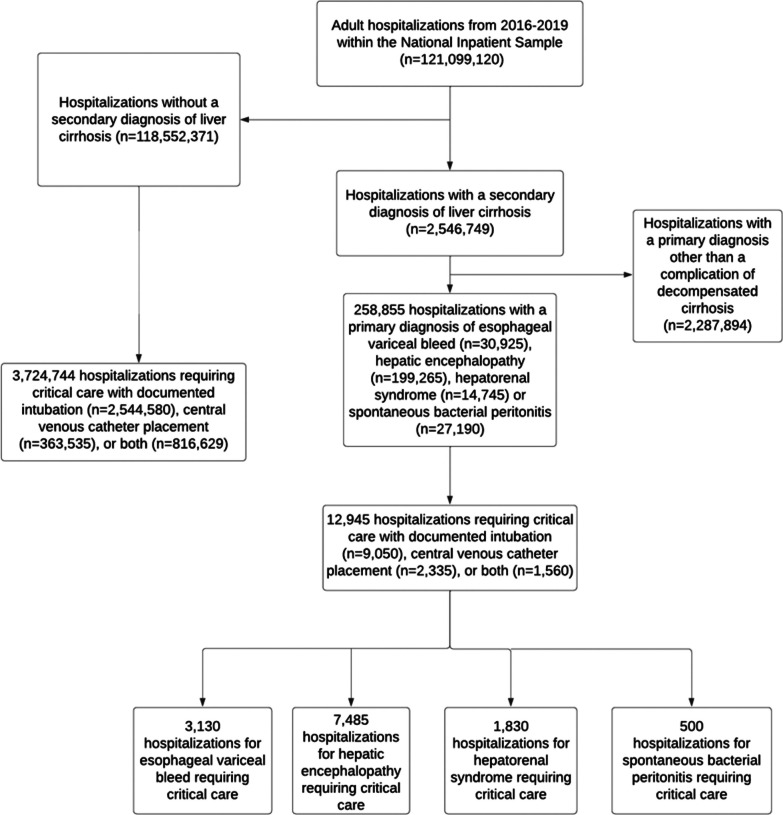
Derivation of a cohort of hospitalizations requiring critical care for complications of decompensated cirrhosis. Derivation of a comparison cohort of hospitalizations requiring critical care in patients without cirrhosis is also shown

Hospitalizations 2016–2019 requiring critical care with a primary diagnosis of esophageal variceal hemorrhage, hepatic encephalopathy, hepatorenal syndrome, or spontaneous bacteria peritonitis were assessed (Fig. 1). A secondary diagnosis of cirrhosis was also an inclusion criterium. These hospitalizations were compared to hospitalizations requiring critical care in patients who did not have a discharge diagnosis of cirrhosis. Delivery of critical care was defined as having received respiratory intubation, central venous catheter placement or both interventions. All principal diagnoses, secondary diagnoses, and procedures were defined by ICD-10 diagnostic and procedural codes (Table S1). Baseline demographic and clinical data along with hospital-level data were evaluated for each hospitalization with specifically assessed variables found in Table 1.

**Table 1:** Demographic and clinical characteristics of hospitalizations requiring critical care for complications of decompensated cirrhosis

Variable	Hospitalizations for decompensated cirrhosis complications	Hospitalizations in patients without cirrhosis	*p* value
Sample size	12,945	3,724,744	
Mean age, years (SD)	58.0 (11.2)	62.5 (16.9)	< 0.001**
Female, %	38.2	46.1	< 0.001**
Race, %			
White	63.8	64.2	0.610
Black	11.1	19.1	< 0.001**
Hispanic	17.8	10.2	< 0.001**
Asian or Pacific Islander	2.9	2.7	0.695
Native American	1.2	0.7	0.001*
Other	3.2	3.1	0.808
Alcohol use, %	42.8	8.8	< 0.001**
Hepatitis C virus, %	23.7	2.6	< 0.001**
Hepatitis B virus, %	3.4	0.4	< 0.001**
Autoimmune hepatitis, %	1.3	0.0	< 0.001**
Primary biliary cholangitis, %	1.7	0.0	< 0.001**
Primary sclerosing cholangitis, %	0.6	0.2	< 0.001**
Diabetes, %	14.3	10.6	< 0.001**
Obesity, %	13.7	17.7	< 0.001**
Chronic kidney disease %	30.6	29.2	0.105
Human immunodeficiency virus, %	1.2	0.9	0.176
Hepatocellular carcinoma, %	6.1	0.1	< 0.001**
Palliative care encounter, %	21.4	14.5	< 0.001**
Primary payer, %			
Medicare	40.5	59.4	< 0.001**
Medicaid	30.0	17.2	< 0.001**
Private	22.8	18.8	< 0.001**
Self-pay	6.7	4.6	< 0.001**
Hospital region, %			0.129
Northeast	17.3	16.5	0.248
Midwest	20.6	20.3	0.668
South	41.1	43.4	0.026*
West	21.0	19.8	0.174
Rural hospital, %	5.8	6.7	0.060

### Outcomes

Clinical outcomes included mortality, the use of renal replacement therapy (RRT) and the use of cardiopulmonary resuscitation (CPR). Outcomes were described for the combined cohort (all four primary diagnoses) and separately for the four unique primary diagnoses; esophageal variceal hemorrhage, hepatic encephalopathy, hepatorenal syndrome and spontaneous bacterial peritonitis. The prevalence of RRT was described and mortality was assessed in those who received RRT and those who did not receive RRT. Similarly, the prevalence of CPR was described as was mortality in those who underwent CPR.

### Statistical analysis

The NIS incorporates a self-weighted sample design that is intended to ensure that results are representative of the U.S. inpatient population. The sampling is a stratified systematic random sample of hospitalizations. Weighted results were created using the standard procedures outlined by HCUP and all reported results are weighted. Continuous variables were described with means (with the standard deviation provided for age) and categorical variables, including baseline clinical and hospital level data along with clinical outcomes, were described using proportions. The means of continuous variables were compared with the Student’s t-test. Proportions were compared using chi-square. Hospitalizations with missing variables were not excluded from the study, however, they were not included in proportion calculations that involved the missing variables. STATA, version 17.0 was used for all statistical computations.

## Results

### Study demographic and clinical characteristics

A total of 12,945 hospitalizations for complications of decompensated cirrhosis were included in the study. The mean age of patients was 58 years (SD = 11.2 years) and 38% of the hospitalizations were for female patients. The majority of the cohort was white (63.8%) with other races comprising 36.2% of the cohort (Table 1). Hepatic encephalopathy was the most common primary diagnosis, accounting for 7485 hospitalizations (57.8%). There were 3130 hospitalizations (24.2%) for esophageal variceal hemorrhage, 1,830 (14.1%) for hepatorenal syndrome, and 500 (3.9%) hospitalizations for spontaneous bacterial peritonitis. Intubation was the sole criterium met for critical care in 69.9% of the hospitalizations while 18.0% of the cohort underwent central venous catheter placement without intubation and 12.1% underwent both procedures. There were 3,724,744 hospitalizations requiring critical care in patients without cirrhosis during the same period of time. In comparison to hospitalizations for patients without cirrhosis, hospitalizations for complications of decompensated cirrhosis tended to occur in younger patients (mean age 58.0 vs. 62.5 years, *p* < 0.001) and these patients were more likely to be male (61.8% vs. 53.9%, *p* < 0.001). Additionally, palliative care encounters were more common in hospitalizations for decompensated cirrhosis complications compared to hospitalizations for patients without cirrhosis (21.4% vs. 14.5%, *p* < 0.001).

### In-hospital mortality

**Fig. 2:**
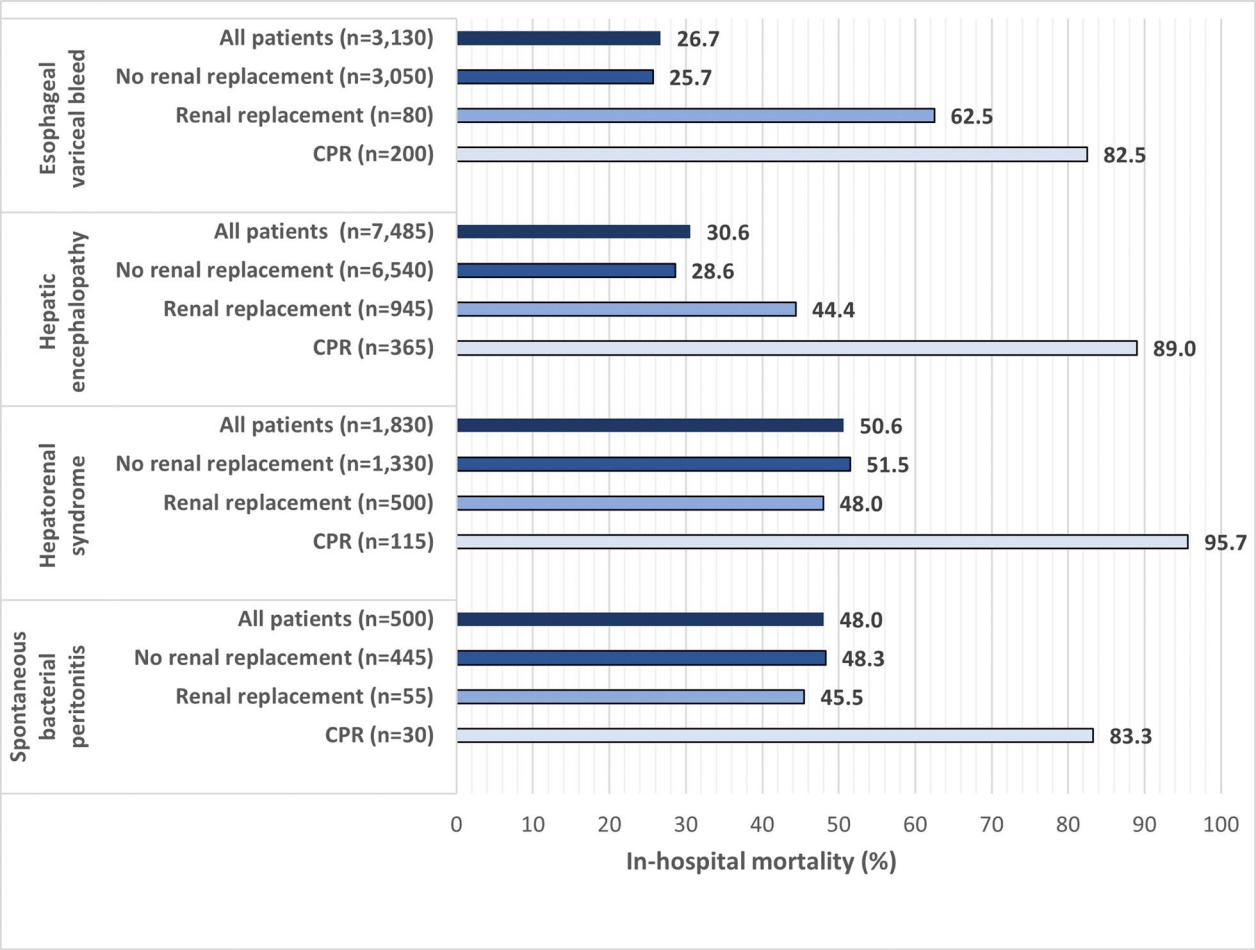
In-hospital mortality for decompensated cirrhosis complications requiring critical care. Results are stratified by primary diagnosis for the hospitalization and the need for renal replacement therapy and cardiopulmonary resuscitation

In total, 4290 deaths were recorded in patients with complications of decompensated cirrhosis while 990,205 deaths were recorded in patients without cirrhosis. In-hospital mortality for complications of decompensated cirrhosis for all patients was 33.1%, which was significantly higher (*p* < 0.001) than the 26.6% mortality noted in hospitalizations for patients without a history of cirrhosis (Table 2). Mortality in complications of decompensated cirrhosis ranged from 26.7% in hospitalizations for esophageal variceal hemorrhage to 50.6% in hospitalizations for hepatorenal syndrome (Fig. 2).

**Table 2:** Clinical outcomes of hospitalizations requiring critical care in patients with complications of decompensated cirrhosis

	Hospitalizations for decompensated cirrhosis complications (n = 12,945)	Hospitalizations in patients without cirrhosis (n = 3,724,744)	*p* value
Renal replacement therapy, %	12.2	8.4	< 0.001**
Cardiopulmonary resuscitation, %	5.5	8.4	< 0.001**
Mortality, %			
All hospitalizations	33.1	26.6	< 0.001**
Received renal replacement therapy	46.5	32.3	< 0.001**
Underwent cardiopulmonary resuscitation	88.0	72.1	< 0.001**

### RRT prevalence and outcomes

RRT was utilized in 1580 (12.2%) hospitalizations for decompensated cirrhosis complications, which was higher compared to hospitalizations in patients without cirrhosis (8.4%) (*p* < 0.001). RRT was most commonly utilized when the primary diagnosis was hepatorenal syndrome (500/1830, 27.3%). In patients who did not undergo RRT (n = 11,365), overall mortality was 31.3% and esophageal variceal bleed remained the condition with the lowest mortality (25.7%) while hepatorenal syndrome had the highest mortality (51.5%). Mortality in hospitalizations for complications of decompensated cirrhosis that received RRT was 46.5% and ranged from 44.4% in cases of hepatic encephalopathy to 62.5% in cases of esophageal variceal hemorrhage. For hospitalizations requiring critical care in patients without cirrhosis, mortality when RRT was utilized was 32.3% which was significantly lower (*p* < 0.001) than mortality in the decompensated cirrhosis cohort.

### CPR prevalence and outcomes

Of the 12,945 hospitalizations for complications of decompensated cirrhosis, CPR was utilized in 710 (5.5%). CPR rates ranged from 4.9% in hospitalizations for hepatic encephalopathy to 6.4% in hospitalizations for esophageal variceal hemorrhage. Death occurred in 88.0% of hospitalizations that included the use of CPR and ranged from 82.5% in cases of esophageal variceal hemorrhage to 95.7% in cases of hepatorenal syndrome. CPR rates were similar between patients who underwent RRT (5.7%) and patients who did not undergo RRT (5.5%, *p* = 0.86). In comparison, hospitalizations for patients without cirrhosis had a higher rate of CPR (8.4%, < 0.001) and lower mortality when CPR was utilized (72.1%, *p* < 0.001).

## Discussion

One-third of patients requiring critical care for complications of decompensated cirrhosis did not survive-to-discharge in our 4-year U.S. nationwide analysis. These results provide generalizable information on critical care outcomes for patients within the U.S. with decompensated cirrhosis. In addition, we demonstrate an increase in mortality in those requiring RRT as well as poor CPR outcomes in this population.

Overall in-hospital mortality for complications of decompensated cirrhosis was increased compared to hospitalizations for patients without cirrhosis and was within the range of previously reported ICU mortality in patients with cirrhosis (29–87%) [4, 8, 12, 13, 17–20, 23, 24]. However, it is noteworthy that at 33.1%, our findings were on the lower end of previously reported mortality and therefore highlight the importance of nationwide large cohorts to better understand generalized outcomes. Prior evaluations of critical care outcomes in patients with cirrhosis have largely used ICU admission as an inclusion criterium without specification to whether or not patients underwent respiratory intubation or central line placement [8, 14, 17–19]. Patients in the ICU requiring respiratory intubation or the placement of a central venous catheter have been shown to have worse outcomes than those who do not require either [25–27]. All subjects in our study underwent respiratory intubation and/or central venous catheter placement. Mortality in our study being on the lower end of previously reported rates despite our inclusion criteria suggests that outcomes are either improving or that previous conclusions were overly pessimistic [2, 8, 10, 13, 28, 29]. Notably, we did include patients who received a liver transplant during the index hospitalizations (n = 400), which is another possible explanation for the relatively low mortality. However, this had relatively little impact in our analysis, as even after excluding those hospitalizations, mortality in our study remained on the lower end of previously reported rates (34.0%).

A large-scale assessment of a combined cohort of patients in Australia and New Zealand found that ICU mortality in those with cirrhosis declined from 44% in 2000 to 29% in 2015 [29]. Another large-scale assessment of patients in the United Kingdom found that mortality in those with cirrhosis admitted to the ICU declined from 58% in 1998 to 46% in 2012 [8]. This trend has been demonstrated in the U.S. as well, with a study by Cheung et al. showing improvement in mortality of intubated patients with cirrhosis from 2005 to 2014 [30]. Advances in the management of complications of cirrhosis, including variceal hemorrhage and hepatorenal syndrome, have been described recently and these, along with improvements in critical care in general, may be responsible for the trend of improvement noted in the literature and supported by our findings [4, 9, 11, 13, 31–33].

An important distinction between our study and previous assessments is that we solely evaluated admissions for complications of decompensated liver disease while many of the previous assessments included ICU admissions for any reason in patients with cirrhosis [8, 18, 19, 29]. This may have also contributed to the relatively low mortality in our study if admission reasons not assessed in our study portend high mortality. Sepsis, in particular, warrants mention as infection accounts for approximately 50% of deaths in patients with cirrhosis [34]. While spontaneous bacterial peritonitis was the only infectious complication we specifically evaluated for, prior studies have identified infection as a common trigger for acute decompensation events with concurrent infection present in 66% of cases of upper gastrointestinal bleeding and 64% of cases of hepatic encephalopathy in patients with cirrhosis [35–37]. It is therefore likely that infection complicated many of the cases we included despite the primary diagnoses being non-infectious complications of cirrhosis. Our method of evaluation, while limited in regard to comparing to prior studies, provides a study design that can be replicated in future studies to assess trends in critical care outcomes specifically for patients with complications of decompensated cirrhosis.

Renal dysfunction is common in hospitalized patients with cirrhosis with prior studies finding that approximately 20% of patients with cirrhosis admitted to the hospital experience renal dysfunction, and up to 50% admitted to the ICU experience renal failure with 20–30% receiving RRT [11, 12, 38, 39]. Our findings are in-line with previous work as we found RRT was utilized more in hospitalizations for patients with cirrhosis compared to those without cirrhosis. RRT was used in 12.2% of hospitalizations for decompensated cirrhosis and mortality was significantly increased in those who required RRT (46.5% vs. 31.3%, *p* < 0.001). Renal failure has previously been identified as a risk factor for mortality in patients with cirrhosis [14, 18, 21, 23, 38]. Indeed, for non-transplant candidate patients with cirrhosis, inpatient mortality after initiating RRT has been estimated to be between 65 and 75% with 6-month mortality as high as 85% [39–43]. Our findings again suggest a decreased prevalence of RRT and mortality in those who receive it, but we are limited by the inability to follow-up patients after discharge. Additionally, we are unable to assess which patients went on to receive a transplant after discharge.

CPR outcomes in patients with cirrhosis have been shown to be worse than in patients with metastatic cancer with previous studies estimating in-hospital mortality rates from 85 to 90% [44–46]. Our study supports these previous findings with an in-hospital mortality rate of 88% which was significantly higher than the in-hospital mortality when cirrhosis was not present. Notably, our study found that outcomes were particularly poor in those with hepatorenal syndrome receiving CPR with 96% not surviving to discharge. Palliative care encounters were increased in hospitalizations for decompensated cirrhosis complications compared to hospitalizations for patients without cirrhosis in our study (21.4% vs. 14.5%, *p* < 0.001), but it is notable that the majority (78.6%) of those critically ill with complications of decompensated cirrhosis do not have a palliative care encounter. The poor CPR outcomes noted in this study further emphasize the need for honest discussions around prognosis, possibly with the assistance of a palliative care professional, to allow patients and families to make informed decisions about their code status.

Significant strengths of our study include the large, nationwide, generalizable sample and our reporting of outcomes stratified by primary admission diagnosis. Inclusion of patients who received a liver transplant during the index hospitalization improves the generalizability of our results. However, we limited our discussion of liver transplantation outcomes in this study as the NIS does not specify whether or not a patient was admitted to a liver transplant center or eventually referred to a transplant center. Other limitations include the reliance on ICD-10 codes, a lack of laboratory data and the inability to provide information on outcomes after hospital discharge which limits our conclusions largely to inpatient outcomes. The lack of laboratory data prevented us from including commonly used prognostic tools for both liver disease and critical care such as Model for End-Stage Liver Disease and Sequential Organ Failure Assessment scores and it limited the ability to fully assess the extent and number of organ failures in individual patients. However, we would note that a previous single center assessment of ICU mortality in patients with cirrhosis found MELD to be a relatively poor prognostic marker while factors such as the need for RRT and intubation, which were included in our study, were more predictive of mortality ^41^. Restricting our definition of critical care to those requiring intubation or a central venous catheter likely excluded many patients who did require ICU admission. Additionally, the inclusion of these procedures within the NIS is contingent upon documentation of them being performed and billing for the procedures and the NIS does not provide the indication for procedures. However, we believe that providing an operational definition of critical care which can be applied to future studies using administrative databases is a balancing strength and will allow for future trend analysis.

## Conclusions

Critical care outcomes may be improving in patients with cirrhosis. The need for RRT may not be as poor of a prognostic marker as previously believed with 53% of those who required it surviving to discharge. CPR outcomes, on the other hand, remain poor with 88% in-hospital mortality. These findings aide in defining trends in critical care outcomes in patients with cirrhosis and can inform prognosis discussions with patients and families, particularly when discussing code status.

### Supplementary Information

Supplementary file1 (DOCX 23 KB)

## Data Availability

The dataset supporting the conclusions of this article is available in the National Inpatient Sample repository, https://hcup-us.ahrq.gov/tech_assist/centdist.jsp.

## Abbreviations

CPR: Cardiopulmonary resuscitation
HCUP: Healthcare cost and utilization project
ICD: International classification of diseases
ICU: Intensive care unit
NIS: National inpatient sample
RRT: Renal replacement therapy
SD: Standard deviation
U.S.: United States
