# Supplementary material for: Critical care outcomes in decompensated cirrhosis: a United States national inpatient sample cross-sectional study
Source: Crit Care. 2024 May 7;28:150. doi: 10.1186/s13054-024-04938-8 (PMC11077702; doi:10.1186/s13054-024-04938-8)
Supplement: Supplementary file 1 — Supplementary file1 (DOCX 23 KB) [file 13054_2024_4938_MOESM1_ESM.docx]

**Critical Care Outcomes in Decompensated Cirrhosis: A United States National Inpatient Sample Analysis**

Authors: Spencer R Goble, Abdellatif S Ismail, Jose D Debes, Thomas M Leventhal

Table of Contents:

Supplementary methods: …………………………………………………………………………. 2

Supplementary results: ……………………………………………………………………………………………………………..3

Table S1. ICD-10 diagnostic procedural codes used to assess critical care outcomes in hospitalizations for complications of decompensated cirrhosis

| Alcohol use | F10*, K70.0, K70.10, K70.30, K70.9 |
| --- | --- |
| Autoimmune hepatitis | K75.4 |
| Central line placement | 06HN33Z, 06HM33Z, 05H633Z, 05H533Z, 05HN33Z, 05HM33Z, 05HM3DZ |
| Chronic kidney disease | N18, N18.1, N18.2, N18.3, N18.4, N18.5, N18.6, N18.7, N18.8, N18.9, N19, R94.4 |
| Cirrhosis | K71.7, K74.3, K74.4, K74.5, K70.30, K70.31, K74.60, K74.69 |
| CPR | 5A12012 |
| Diabetes | E10, E10.1, E10.2, E10.3, E10.4, E10.5, E10.6, E10.7, E10.8, E10.9, E11, E11.1, E11.2, E11.3, E11.4, E11.5, E11.6, E11.7, E11.8, E11.9 |
| Esophageal varices, bleeding | I85.01, I85.11 |
| Esophageal varices, non-bleeding | I85.10, I85.00 |
| HBV | B16.2, B16.9, B18.1, B19.1, B19.10. B19.11 |
| HCV | B17.10, B17.11, B181.2, B19.20, B19.21, Z22.52 |
| Hepatic encephalopathy | K72.91, K72.01, K72.11,  K72.90 |
| Hepatocellular carcinoma | C22.8, C22.0 |
| Hepatorenal syndrome | K76.7 |
| HIV | B20.0, B20.1, B20.2, B20.3, B20.4, B20.5, B20.6, B20.7, B20.8, B20.9, B21.0. B21.1, B21.2, B21.3, B21.7, B21.8, B21.9, B22.0, B22.1, B22.2, B22.7, B23.0, B23.1, B23.2, B23.8, B20, B24, Z21 |
| Intubation | 0BH17EZ, 0BH18EZ |
| Palliative care encounter | Z515 |
| Primary biliary cholangitis | K74.3, K74.5 |
| Primary sclerosing cholangitis | K83.0 |
| Renal replacement therapy | Z992, Z4901, Z4931, 5A1D* |
| Spontaneous bacterial peritonitis | K65.2 |

Table S2. Clinical outcomes of hospitalizations requiring intubation without central venous catheter placement comparing patients with complications of decompensated cirrhosis to patients without cirrhosis

|  | Hospitalizations for decompensated cirrhosis complications (n=9,050) | Hospitalizations in patients without cirrhosis (n=2,544,580) | *P*-Value |
| --- | --- | --- | --- |
| Renal replacement therapy, % | 9.6 | 5.7 | <0.001** |
| Cardiopulmonary resuscitation, % | 5.9 | 9.8 | <0.001** |
| Mortality, % |  |  |  |
| All hospitalizations | 33.3 | 29.4 | <0.001** |
| Received renal replacement therapy | 50.3 | 44.6 | <0.001** |
| Underwent cardiopulmonary resuscitation | 85.8 | 71.9 | <0.001** |

**p* < 0.05, ***p* < 0.001

Table S3. Clinical outcomes of hospitalizations requiring central venous catheter placement without intubation comparing patients with complications of decompensated cirrhosis to patients without cirrhosis

|  | Hospitalizations for decompensated cirrhosis complications (n=2,335) | Hospitalizations in patients without cirrhosis (n=816,629) | *P*-Value |
| --- | --- | --- | --- |
| Renal replacement therapy, % | 19.3 | 14.9 | 0.008* |
| Cardiopulmonary resuscitation, % | 1.2 | 1.8 | 0.425 |
| Mortality, % |  |  |  |
| All hospitalizations | 19.3 | 10.9 | <0.001** |
| Received renal replacement therapy | 25.6 | 10.8 | <0.001** |
| Underwent cardiopulmonary resuscitation | 100.0 | 72.0 | - |

**p* < 0.05, ***p* < 0.001

Table S4. Clinical outcomes of hospitalizations requiring central venous catheter placement and intubation comparing patients with complications of decompensated cirrhosis to patients without cirrhosis

|  | Hospitalizations for decompensated cirrhosis complications (n=1,560) | Hospitalizations in patients without cirrhosis (n=363,535) | *P*-Value |
| --- | --- | --- | --- |
| Renal replacement therapy, % | 17.0 | 13.1 | 0.045* |
| Cardiopulmonary resuscitation, % | 9.6 | 14.1 | 0.024* |
| Mortality, % |  |  |  |
| All hospitalizations | 52.9 | 42.1 | <0.001** |
| Received renal replacement therapy | 69.8 | 49.6 | <0.001** |
| Underwent cardiopulmonary resuscitation | 93.3 | 73.2 | <0.001** |

**p* < 0.05, ***p* < 0.001

Table S5. Clinical outcomes of hospitalizations 2016-2019 requiring central venous catheter placement and/or intubation comparing patients with cirrhosis to patients without cirrhosis

|  | Hospitalizations for patients with cirrhosis (n=186,900) | Hospitalizations in patients without cirrhosis (n=3,724,744) | *P*-Value |
| --- | --- | --- | --- |
| Renal replacement therapy, % | 12.8 | 8.4 | <0.001** |
| Cardiopulmonary resuscitation, % | 7.5 | 8.4 | <0.001** |
| Mortality, % |  |  |  |
| All hospitalizations | 39.8 | 26.6 | <0.001** |
| Received renal replacement therapy | 50.0 | 32.3 | <0.001** |
| Underwent cardiopulmonary resuscitation | 80.9 | 72.1 | <0.001** |

**p* < 0.05, ***p* < 0.001
